# Orthostatic test shows higher systolic blood pressure and sympathetic response in uncomplicated type 1 diabetes patients with normal V̇O_2max_ vs. healthy controls

**DOI:** 10.1007/s10286-024-01094-5

**Published:** 2024-12-17

**Authors:** Samu Sorola, Vesa Hyrylä, Timo Eronen, Saana Kupari, Mika Venojärvi, Heikki Tikkanen, Mika Tarvainen, Harri Lindholm

**Affiliations:** 1 Institute of Biomedicine, Sports and Exercise Medicine, University of Eastern Finland, Kuopio, Finland; 2 Department of Technical Physics, University of Eastern Finland, Kuopio, Finland; 3 Department of Clinical Physiology and Nuclear Medicine, Kuopio University Hospital, Kuopio, Finland

**Keywords:** Type 1 diabetes, Heart rate variability, Deep breathing test, Orthostatic test, Cardiorespiratory fitness

## Abstract

**Purpose:**

Cardiovascular autonomic neuropathy remains underdiagnosed in type 1 diabetes mellitus, posing a risk for severe complications, particularly in patients with lowered V̇O_2max_, compared to controls. This study aimed to determine whether heart rate variability during cardiovascular autonomic reflex tests reveals early signs of cardiovascular autonomic neuropathy in patients with uncomplicated type 1 diabetes mellitus and normal cardiovascular fitness, compared to healthy controls.

**Methods:**

A type 1 diabetes mellitus group (*n* = 14) with no other diagnosed diseases (diabetes duration 15 ± 7 years) and a control group (*n* = 31) underwent deep breathing test, passive orthostatic test, and cardiopulmonary exercise test. Participants were assessed for heart rate variability, heart rate, blood pressure, and V̇O_2max_ (mL/min/kg).

**Results:**

Participant characteristics, including V̇O_2max_ (mL/min/kg), showed no significant differences. The type 1 diabetes mellitus group had higher systolic blood pressure during the supine phase of the orthostatic test than healthy controls (131.6 ± 14.7 mmHg vs. 122.4 ± 10.8 mmHg, *p* = 0.022). After 5 mins in the upright position, systolic blood pressure (132.2 ± 20.6 mmHg vs. 118.7 ± 11.7 mmHg, *p* = 0.036), heart rate (85 (76; 89) bpm vs. 75 (72; 83) bpm, *p* = 0.013), and the root mean square of successive RR interval differences (20.22 (11.22; 27.42) vs. 27.11 (19.90; 35.52), *p* = 0.033) were significantly different compared to controls.

**Conclusion:**

Patients with uncomplicated type 1 diabetes mellitus, despite having normal cardiorespiratory fitness, exhibited higher systolic pressure and greater sympathetic activation in orthostatic tests, suggesting subclinically altered cardiovascular autonomic function.

**Supplementary Information:**

The online version contains supplementary material available at 10.1007/s10286-024-01094-5.

## Introduction

Type 1 diabetes mellitus (T1DM) is a chronic autoimmune disease caused by the destruction of pancreatic β-cells, leading to the complete cessation of insulin production. This cessation of insulin production results in an inability to regulate blood glucose levels, necessitating lifelong insulin therapy [1]. Chronically elevated blood glucose levels (i.e. hyperglycaemia) in T1DM lead to complications in the cardiovascular and nervous systems. One such complication is diabetes-associated cardiovascular autonomic neuropathy (CAN), where hyperglycaemia contributes to nerve fibre and blood vessel damage [2]. CAN significantly impairs cardiovascular and autonomic nervous system functions and is known to progress from subclinical stages to severe manifestations that may include myocardial ischaemia and stroke [3].

Although CAN is a severe condition in patients with T1DM, it is underdiagnosed and lacks standardized diagnostic criteria [4]. The subclinical stages of CAN can be identified with cardiovascular autonomic reflex tests (CART). CART batteries include the assessment of cardiovascular autonomic responses to physical stressors such as exercise, deep breathing, and postural changes [5]. In addition, cardiac autonomic function can be assessed by measuring the variation in time intervals between successive heartbeats from electrocardiogram (ECG) data, known as heart rate variability (HRV). HRV can be incorporated into a CART battery, as it is a non-invasive and cost-effective method, and has also been suggested to be more sensitive in detecting early signs of CAN during supine rest in T1DM than other traditional tests [6].

At the subclinical stage of CAN, patients with T1DM often exhibit reduced resting HRV, indicating impaired parasympathetic nervous system function. As CAN progresses to the clinical stage, this impairment becomes more evident, with blunted HRV responses to breathing tests and resting tachycardia, signalling both parasympathetic impairment and sympathetic overactivity [7]. Additionally, patients may develop orthostatic hypotension, reflecting a compromised cardiovascular autonomic response to postural changes, often manifesting as exercise intolerance [4].

Exercise intolerance in T1DM is further elucidated through cardiopulmonary exercise testing (CPET), which measures maximal oxygen consumption (V̇O_2max_). V̇O_2max_ is an essential indicator of an individual’s cardiorespiratory fitness. Patients with T1DM typically exhibit lower V̇O_2max_, attributable to cardiovascular complications and altered muscle metabolism. Furthermore, poor blood glucose control in T1DM has been linked to reduced V̇O_2max_ [8]. V̇O_2max_ is related to heart rate (HR) and HRV [9, 10]. In particular, HRV time-domain measurements show a positive correlation with aerobic fitness and health [11]. Additionally, regular physical activity has been shown to increase HRV [12].

Intriguingly, research indicates that patients with uncomplicated T1DM (no other chronic diseases excluding T1DM) display subclinical signs of CAN, with higher resting HR and lower V̇O_2max_ compared to healthy controls (CON), alongside reduced HRV [13]. Our aim is to investigate whether patients with uncomplicated T1DM, who maintain V̇O_2max_ levels comparable to the CON group, still show subclinical signs of CAN in CART assessments of deep breathing and orthostatic tests and incorporated HRV indices. Our interest is applicability—specifically, using simple, easy-to-use HRV indices that are accurate in short-term recording periods and robust against changes in respiratory rate, as we will be investigating results from both paced and self-paced breathing.

## Methods

The data used in this study were part of the Effects of Exercise and Stress on Glucose Metabolism in Type 1 Diabetes (DIAMES) project, which was carried out at the University of Eastern Finland during 2021–2022. The DIAMES study was approved by the ethics committee of the Northern Savo Hospital District, Kuopio, Finland (reference number 409/2019). The project follows the Helsinki Declaration in its latest version, good clinical practice, and the General Data Protection Regulation (GDPR). All study participants signed an informed consent prior to entering the study procedures. There was no payment for participation in the study.

### Study participants

Initially, the participants included patients with T1DM (*n* = 18) and healthy controls (CON) (*n* = 35) who completed the tests. A total of eight participants were excluded from the study. Three participants in the T1DM group were taking blood pressure medication. Additionally, one T1DM participant was excluded as a result of aberrant HRV data that could not be adequately adjusted for in any of the statistical analyses. In the CON group, two participants had irregular heart rhythms accounting for more than 5% of the recorded data, while two others did not complete all six breathing cycles during the deep breathing test. After these exclusions, the T1DM group had 14 participants, and the CON group had 31 participants.

The recruitment of participants was carried out by the University of Eastern Finland in collaboration with the endocrinology and diabetology outpatient clinic at Kuopio University Hospital. The study was advertised locally through news publications, associations, the university campus, and social media channels.

Inclusion criteria for the study group were a confirmed diagnosis of T1DM requiring insulin therapy, with a disease duration of 3–25 years, age between 18 and 50 years, and a body mass index (BMI) of 18–35 kg/m^2^. Participants were excluded if they had any diagnosed neuropathy, nephropathy, retinopathy, asthma, hypertension, or other chronic diseases. The inclusion criteria for the control group was age, gender, and BMI matching with the T1DM group, and no diagnosed chronic illnesses (no medication-requiring illnesses, age 18–50 years, and BMI 18–35 kg/m^2^). The exclusion criteria for both groups were (1) known coronary artery disease or history of heart attack, (2) lower limb amputation, (3) pacemaker, (4) renal insufficiency, which is confirmed by eGFR < 60 ml/min, or rapidly progressing microalbuminuria and oedema, (5) nephrotic syndrome, (6) medications that affect the cardiovascular system, (7) severe asthma requiring continuous medication, (8) smoking, or (9) physical inability to exercise. With these criteria, it was discovered after CPET that both the T1DM and CON groups were V̇O_2max_-matched. Two participants in the T1DM group were on mild cholesterol medication (rosuvastatin 5 mg). One participant in the T1DM group self-reported 2–3 hypoglycaemic events within the 12 months prior to the test day. The participant reported that these events did not have an effect at the time of testing. Half of the participants in the T1DM group (*n* = 7) used multiple daily injections (MDI), while the other half used an insulin pump.

### Experimental protocol

**Fig. 1:**
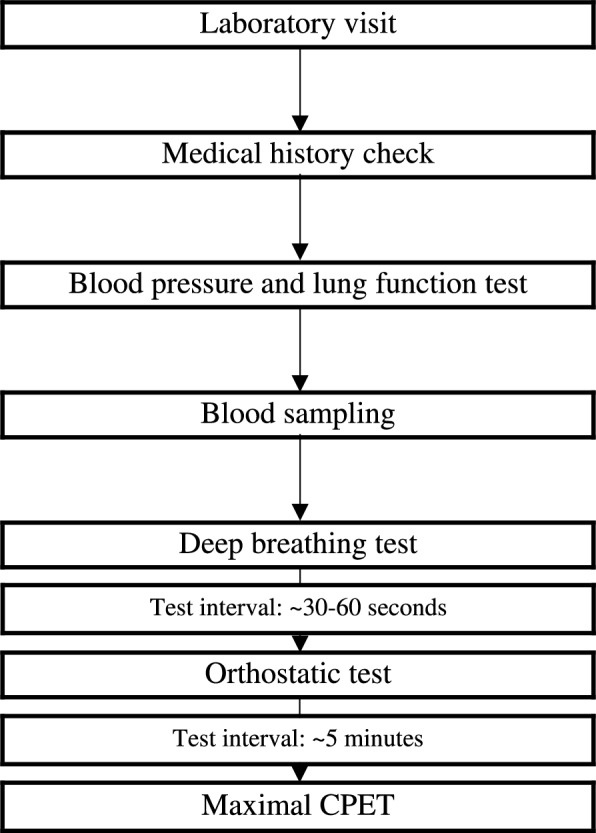
Test protocol flowchart

The experimental protocol is presented in Fig. 1. Participants were required to be healthy for at least 2 weeks before the laboratory visit and must not have experienced any severe hypoglycaemia (blood glucose < 2.9 mmol/L) in the 24 h prior to the visit. In addition, participants were instructed to abstain from smoking, caffeine, and alcohol consumption for 12 h, and to avoid heavy exercise for 24 h before the tests. On the test day, participants were instructed to consume a normal breakfast and a small lunch if necessary. The tests were conducted between 7 AM and 2 PM. Although individuals with an asthma diagnosis were excluded, some participants may still use bronchodilators without asthma diagnosis; thus, every participant was reminded that the use of bronchodilators was not allowed before or during the tests. The self-reported physical activity questionnaire surveyed the number of hours of physical activity per week.

All tests were conducted at the Human Measurement and Analysis (HUMEA) physiology laboratory within the University of Eastern Finland. Before the actual tests, a series of medical screening tests were conducted, including a medical history check, blood pressure measurement, lung function assessment with spirometry, and a fingertip blood sample from T1DM participants to ensure blood glucose levels were within 5–13.9 mmol/L. Blood glucose levels were tested before and after each test, following applicable standards [14]. If no health obstacles were found during the initial measurements, blood samples were drawn from the brachial vein and collected into EDTA tubes for haemoglobin A1c (HbA1c). HbA1c was determined according to the manufacturer’s instructions (cobas 8000 (c 702) analyser, Roche Diagnostics, Basel, Switzerland).

In the CART assessments, ECG recordings (MP150, two ECG100C, Biopac Systems, Inc., Goleta, CA, USA) were collected from the participants, and ECG-derived respiration (EDR) was used for the respiratory rate analysis. In the deep breathing test, participants first rested for 5 min in a sitting position. Participants were then instructed to lie in a supine position and perform six deep breathing cycles within 1 min by following a guided breathing pattern (inhale followed by exhale, respiratory rate 6 breaths/min or 0.1 Hz), and to breathe through their nose.

The passive orthostatic test started approximately 30–60 s after the deep breathing test and involved 5 min of supine rest, followed by a 5-s table tilt and 5 min of rest in the upright position (70°). Arm blood pressure was measured (HEM-907, Omron Healthcare Europe B.V., Hoofddorp, the Netherlands) (1) at the end of the 5 min of supine rest (just before the table tilt), (2) immediately after the 5-s tilt phase, and (3) at the upright position at the 1-min, 3-min, and 5-min marks after the tilt.

The maximal CPET started approximately 5 min after the orthostatic test and was performed using a cycle ergometer (Ergoline Ergoselect 200K, ergoline GmbH, Bitz, Germany), with the workload increasing every 3 min by 35 W for men and 25 W for women. The participants continued to cycle until clear indications of a performance plateau were observed. Respiratory gases were recorded (Metamax model 3b-r2, Cortex-medical, Leipzig, Germany) to assess participants’ V̇O_2max_.

### HRV analysis

In the deep breathing test, HRV was analysed as the mean HRV over the entire 1-min test duration. In the orthostatic test, resting HRV was analysed as the mean HRV from the 5-min resting period just before tilting the table. The 5-min upright period mean HRV analysis started 30 s after tilting the table. This 30-s wait period was used to minimize the effects of the sudden change in body position, ensuring that the measurement captured the steady-state condition after tilting rather than the immediate response to it.

HRV analysis included time-domain measures of root mean square of successive RR interval differences (RMSSD), standard deviation of NN intervals (SDNN), non-linear measures of Poincaré plot standard deviation perpendicular to the line of identity (SD1), Poincaré plot standard deviation along the line of identity (SD2), and SD2 to SD1 ratio (SD2/SD1), and lastly parasympathetic nervous system (PNS) index, which is computed from values of mean RR interval, RMSSD, and SD1 in Kubios HRV analysis software. In addition, EDR estimates was used to assess respiratory rate during tilt testing. EDR was calculated using Kubios HRV software by analysing respiration-induced changes in the ECG amplitude and the RR interval time series data. The E/I ratio, defined as the maximum RR interval during exhalation divided by the minimum RR interval during inhalation, averaged over the six breathing cycles, was computed from the deep breathing test. All HRV data were analysed with Kubios HRV Scientific 4.1 (Kubios Oy, Finland).

Frequency-domain measures of HRV were not used in this study because the respiratory rate in the deep breathing challenge falls into the LF band by design, and therefore, the division of HRV into low frequency (LF: 0.04–0.15 Hz) and high frequency (HF: 0.15–0.4 Hz) bands is not appropriate. The same applies partly to the supine rest period of the tilt table test, because some participants (the slow breathers) had respiratory rates falling inside the LF band or between the LF and HF bands. Reliable frequency-domain analysis of HRV requires that participants’ respiratory rate is within the HF band [11, 15]. There are also controversies regarding the interpretation of LF power, which has been suggested to be a measure of sympathetic activity [16, 17]. However, more recent evidence suggests that LF power reflects baroreceptor function, which primarily modulates parasympathetic activity under resting conditions [18–21].

HRV parameters such as LF power, LF/HF ratio, SD2, and the SD1/SD2 ratio are suggested to require at least 5-min recordings for accuracy [22]. Therefore, the deep breathing HRV analysis does not include these indices, as they are unsuitable for 1-min recordings.

### Statistical analysis

All data were checked for normality using histograms, QQ plots, and the Shapiro–Wilk test (*n* < 50). Mean HR, RR, EDR, blood pressure variables, and baseline participant characteristics were normally distributed; thus, an independent samples *t* test for two-group comparisons and a paired samples *t* test for within-group testing were selected. Assumptions of adequate sample sizes, normal distribution, and homogeneity of variances (Levene’s test) were violated for self-reported physical activity (h), the E/I ratio, and HRV indices, making parametric tests invalid. Some of the HRV variables were still not normally distributed after log transformations. Consequently, the non-parametric Mann–Whitney *U* test for two-group comparisons and the Wilcoxon signed-rank test for within-group comparisons were selected. Results are presented as medians, interquartile ranges (25th and 75th percentiles), and 95% confidence intervals (CIs) for the median differences. Median differences, along their 95% Cis, were calculated using the Hodges–Lehmann estimator. Adjustments for multiple comparisons were not made because each variable was tested separately between the two groups or within the groups independently. Both mean and median differences are presented with 95% CIs and are considered statistically significant if the *p* value is < 0.05 and the null hypothesis does not fall within the 95% CI. All statistical procedures were carried out using IBM SPSS (Version 27.0.1.0 Microsoft Windows, IBM Corporation, Armonk, New York).

## Results

### Characteristics of study participants

A total of 14 T1DM participants and 31 CON participants were eligible for the study. The characteristics of the study participants are summarized in Table 1. The groups were matched for sex, age, mass, height, BMI, self-reported physical activity, and V̇O_2max_ (mL/min/kg). The mean time from T1DM diagnosis was 15 years. The mean HbA1c (%) for the T1DM participants was 7.5 ± 0.7.

**Table 1:** Participant characteristics

Variable	T1DM (*n* = 14)	CON (*n* = 31)	*p* value
Women/men ratio	7:7	15:16	0.920^a^
Age (years)	33 ± 9	33 ± 7	0.943
Weight (kg)	74.6 ± 11.3	73.4 ± 11.7	0.735
Height (cm)	174.4 ± 8.6	174.0 ± 9.3	0.878
BMI (kg/m^2^)	24.4 ± 2.5	24.2 ± 3.1	0.820
Self-reported physical activity (h/week)	5.3 (4.9; 9)	6.5 (4.4; 9.1)	0.768^b^
V̇O_2max_ (mL/min/kg)	39.6 ± 8	40.2 ± 7.6	0.819
HbA1c (mmol/mol)	58.3 ± 7.6	31.4 ± 2.4	< 0.001*
HbA1c (%)	7.5 ± 0.7	5 ± 0.2	< 0.001*
T1DM duration (years)	15 ± 7		

Values are presented as mean ± SD or median (25th; 75th)

*BMI* body mass index, *HbA1c* glycated haemoglobin

^a^Chi-squared test used for significance testing

^b^Mann–Whitney *U* test used for significance testing

*Significant factors (*p* < 0.05)

### Deep breathing test results

The deep breathing test results are presented in Table 2. No significant differences were found in HRV, HR, or E/I ratio.

**Table 2:** Deep breathing results

Variable	T1DM (*n* = 14)	CON (*n* = 31)	Difference, 95% CI	*p* value
HR (beat/min)	71 (67; 79)	67 (60; 75)	5 (− 1.0, 11.8)	0.082
RR (ms)	839.2 (759.8; 890.8)	897.6 (800.5; 1003.9)	− 5.4 (− 147.6, 11.5)	0.082
RMSSD (ms)	74.04 (27.95; 84.66)	55.61 (34.54; 80.76)	− 0.11 (− 29.61, 25.18)	0.980
SDNN (ms)	106.47 (50.14; 115.43)	91.19 (54.66; 118.86)	1.62 (− 36.90, 24.31)	0.961
SD1 (ms)	52.74 (19.90; 60.29)	39.57 (24.60; 57.64)	− 0.01 (− 21.07, 17.96)	1.0
PNS index	0.25 (− 1.09; 0.76)	− 0.02 (− 0.69; 1.61)	− 0.30 (− 1.39, 0.64)	0.508
E/I ratio	1.40 (1.21; 1.44)	1.34 (1.18; 1.44)	− 0.005 (− 0.153, 0.102)	0.941

Values are presented as medians (25th; 75th)

Median differences are presented as Hodges–Lehmann estimates with 95% CI

### Orthostatic test HRV results

**Fig. 2:**
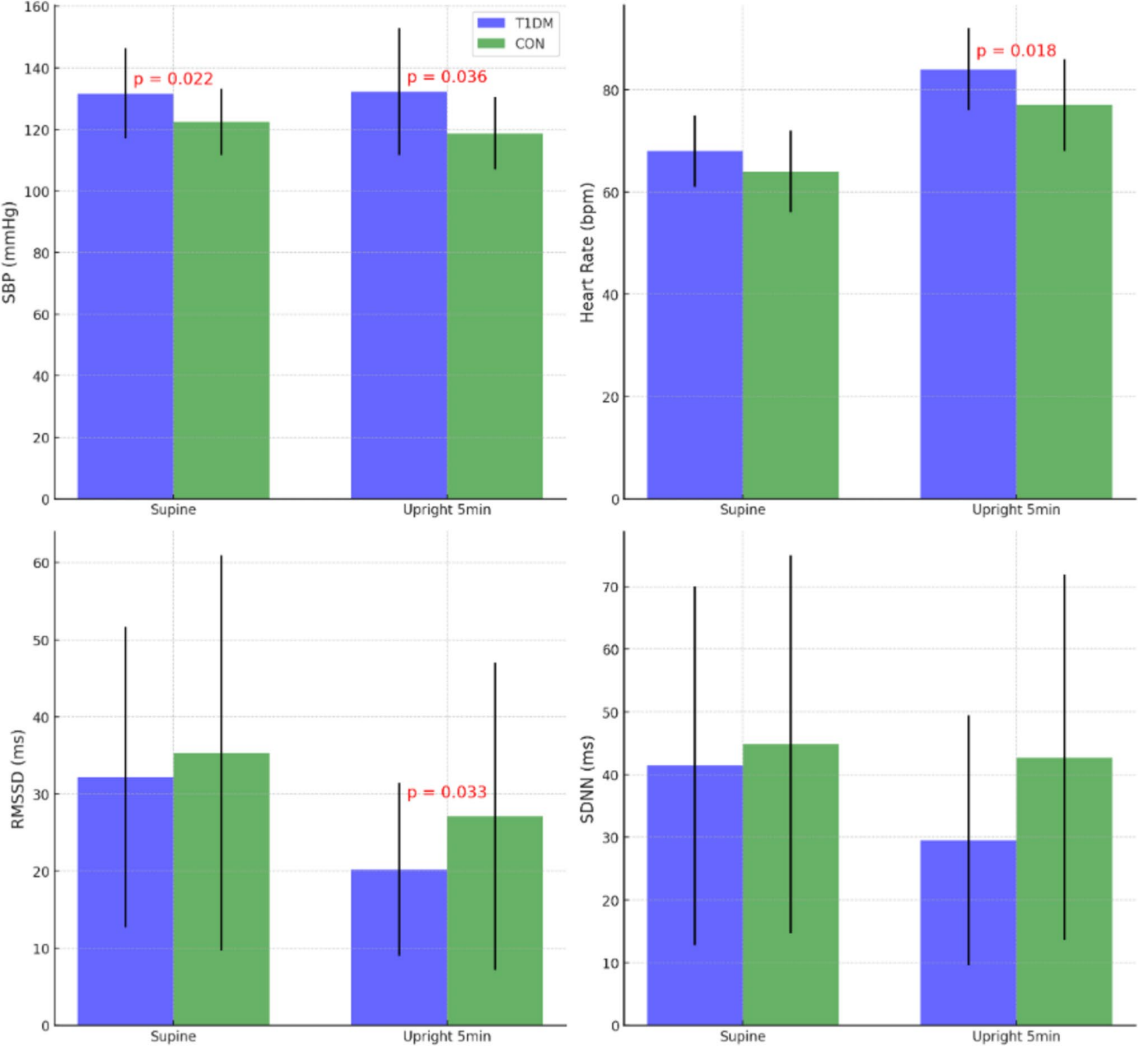
HRV, HR and SBP: T1DM vs. CON groups (supine and upright 5 min)

The orthostatic test HRV results between and within groups are presented in Table 3 and Fig. 2. The between-group comparison of the supine position showed no significant differences in any variable. In the upright position, the T1DM group had significantly lower RR interval, RMSSD, and SD1, and significantly higher HR compared to the CON group, while other HRV indices were not significantly different. EDR was not significantly different between the groups. Within the T1DM group, the comparison from supine to upright positions revealed that all HRV indices and RR interval significantly decreased, while HR significantly increased. In the CON group, SDNN and SD2 did not significantly change, whereas all other HRV indices significantly decreased. EDR did not significantly change from supine to upright in either group.

**Table 3:** Orthostatic test HRV results

Variable	Position	T1DM (*n* = 14)			CON (*n* = 31)			Between group	
		Median	Difference, 95% CI	*P*	Median	Difference, 95% CI	*P*	Difference, 95% CI	*P*
HR (beat/min)	Supine	68 (62; 74)			63 (57; 70)			4 (− 1.1, 9.2)	0.122
	Upright	85 (76; 89)	15 (10.7, 21.2)	< 0.001*	75 (72; 83)	13 (10.4, 15.8)	< 0.001*	7 (1.3, 13.4)	0.013*
RR (ms)	Supine	881.4 (812.9; 967.1)			959.8 (857.0; 1045.8)			− 59.8 (− 128.59, 16.16)	0.121
	Upright	703.0 (672.2; 789.2)	− 165.0 (− 225.7, − 112.3)	< 0.001*	802.4 (720.8; 831.0)	− 161.5 (− 200.5, − 121.9)	< 0.001*	− 65.10 (− 125.50, − 12.80)	0.013*
RMSSD (ms)	Supine	32.17 (19.49; 57.44)			35.33 (25.65; 66.27)			− 3.16 (− 28.61, 19.63)	0.405
	Upright	20.22 (11.22; 27.42)	− 17.46 (− 33.55, − 5.88)	0.004*	27.11 (19.90; 35.52)	− 16.40 (− 24.55, − 8.42)	< 0.001*	− 6.89 (− 15.96, 0.90)	0.033*
SDNN (ms)	Supine	41.44 (28.68; 49.85)			44.87 (30.18; 62.31)			− 4.70 (− 18.27, 8.02)	0.462
	Upright	29.51 (19.92; 45.56)	− 8.81 (− 20.14, − 1.77)	0.019*	42.75 (29.12; 53.89)	− 5.40 (− 11.63, 0.30)	0.063	− 10.04 (− 19.79, 0.56)	0.066
SD1 (ms)	Supine	22.78 (13.80; 40.69)			25.02 (18.17; 46.94)			− 3.32 (− 14.12, 6.85)	0.405
	Upright	14.32 (7.94; 19.41)	− 12.34 (− 23.77, − 4.17)	0.004*	19.20 (14.09; 25.16)	− 11.61 (− 17.40, − 5.97)	< 0.001*	− 5.53 (− 9.62, − 0.46)	0.033*
SD2 (ms)	Supine	50.64 (34.78; 59.57)			56.63 (38.49; 73.00)			− 6.00 (− 21.34, 8.10)	0.377
	Upright	39.72 (26.30; 62.16)	− 8.26 (− 20.97, − 1.07)	0.035*	54.61 (39.08; 71.94)	− 3.04 (− 10.36, 4.00)	0.410	− 13.50 (− 26.34, 1.01)	0.078
SD2/SD1 ratio	Supine	2.13 (1.46; 2.98)			2.00 (1.45; 2.34)			0.11 (− 0.33, 0.74)	0.492
	Upright	3.06 (2.68; 3.63)	0.98 (0.49, 1.18)	0.002*	3.18 (2.47; 3.44)	1.00 (0.82, 1.20)	< 0.001*	0.08 (− 0.35, 0.52)	0.713
PNS index	Supine	− 0.44 (− 1.06; 0.80)			0.03 (− 0.55; 1.12)			− 0.38 (− 1.14, 0.39)	0.220
	Upright	− 1.56 (− 2.04; − 1.35)	− 1.21 (− 2.10, − 0.76)	< 0.001*	− 1.08 (− 1.38; − 0.64)	− 1.26 (− 1.65, − 0.90)	< 0.001*	− 0.51 (− 0.89, − 0.17)	0.006*
EDR (Hz)	Supine	0.17 (0.13; 0.23)			0.19 (0.14; 0.24)			− 0.02 (− 0.06, 0.02)	0.377
	Upright	0.19 (0.15; 0.26)	0.02 (− 0.01, 0.05)	0.133	0.22 (0.16; 0.26)	0.01 (− 0.003, 0.03)	0.096	− 0.01 (− 0.05, 0.03)	0.713

Values are presented as medians (25th; 75th)

Median differences are presented as Hodges–Lehmann estimates with 95% CI

*Significant factors (*p* < 0.05)

### Orthostatic test blood pressure results

The orthostatic test blood pressure results between and within groups are presented in Table 4 and Fig. 2. The between-group comparison of supine position results showed that the T1DM group had significantly higher systolic blood pressure (SBP) and mean arterial pressure (MAP) than the CON group. In the upright position, the T1DM group had significantly higher SBP at the 1-min and 5-min marks. Within the T1DM group, results at the 1-, 3-, and 5-min marks showed a significant increase in diastolic blood pressure (DBP) and MAP, whereas SBP did not significantly change. In the CON group, DBP and MAP significantly increased, while SBP significantly decreased at all time points except the 3-min mark.

**Table 4:** Orthostatic test blood pressure results

Position/time	Variable (mmHg)	T1DM (*n* = 14)			CON (*n* = 31)			Between group	
		Mean ± SD	Difference, 95% CI	*P*	Mean ± SD	Difference, 95% CI	*P*	Difference, 95% CI	*P*
Supine									
	SBP	131.6 ± 14.7			122.4 ± 10.8			9.3 (1.4, 17.1)	0.022*
	DBP	75.6 ± 9.7			71.4 ± 7.4			4.3 (− 1.0, 9.6)	0.111
	MAP	94.3 ± 10.3			88.4 ± 7.0			5.9 (0.7, 11.3)	0.028*
Upright 1 min									
	SBP	130.5 ± 16.4	− 1.1 (− 6.5, 4.2)	0.655	119.0 ± 12.5	− 3.4 (− 6.0, − 0.7)	0.016*	11.5 (2.5, 20.4)	0.013*
	DBP	82.7 ± 12.8	7.1 (3.5, 10.6)	0.001*	77.4 ± 7.7	6.0 (4.2, 7.8)	< 0.001*	5.4 (− 2.4, 13.2)	0.165
	MAP	98.6 ± 12.7	4.3 (1.0, 7.6)	0.014*	91.2 ± 8.1	2.8 (1.5, 4.3)	< 0.001*	7.4 (− 0.3, 15.1)	0.060
3 min									
	SBP	130.1 ± 22.2	− 1.6 (− 7.3, 4.1)	0.561	120.4 ± 10.3	− 2 (− 5.2, 1.2)	0.221	9.7 (− 3.5, 22.9)	0.140
	DBP	84.2 ± 12.9	8.6 (5.4, 11.7)	< 0.001*	78.3 ± 8.2	6.9 (5.0, 8.9)	< 0.001*	5.9 (− 2.0, 13.8)	0.132
	MAP	99.5 ± 14.6	5.2 (1.9, 8.4)	0.004*	92.3 ± 8.0	4 3.9 (1.1, 6.8)	0.003*	7.2 (− 1.6, 16.0)	0.103
5 min									
	SBP	132.2 ± 20.6	0.6 (4.1, − 5.2)	0.795	118.7 ± 11.7	− 3.7 (− 6.0, − 1.3)	0.004*	13.5 (1.0, 25.9)	0.036*
	DBP	82.2 ± 12.1	6.6 (2.4, 10.8)	0.005*	78.5 ± 7.3	7.1 (5.3, 9.1)	< 0.001*	3.7 (− 3.6, 11.0)	0.302
	MAP	98.9 ± 13.6	4.6 (0.9, 8.2)	0.018*	91.9 ± 7.5	3.5 (2.2, 5.0)	< 0.001*	7.0 (− 1.2, 15.2)	0.091

The within-group mean differences represent changes from the baseline supine values to the respective upright measurements at each time point

Values are presented as mean ± SD, with the mean difference based on central tendency and 95% CI

*Significant factors (*p* < 0.05)

## Discussion

Our study aimed to investigate potential cardiac autonomic changes in patients with uncomplicated T1DM, who were matched with the CON group for V̇O_2max_ (mL/min/kg), age, sex, self-reported physical activity, and BMI. We compared HR, HRV, and blood pressure during supine deep breathing and orthostatic passive head-up tilt tests within and between these groups. In the deep breathing test, no notable differences were observed between the T1DM and CON groups. In the supine position of the orthostatic test, the T1DM group had significantly higher SBP and MAP compared to the CON group. In the upright position, SBP remained significantly higher in the T1DM group and did not significantly decrease, in contrast to the CON group. In the upright position, the T1DM group also had significantly higher HR and lower RMSSD, SD1, and PNS index, indicating greater parasympathetic withdrawal. Additionally, SDNN and SD2, which indicate overall sympathetic and parasympathetic activity, were lower in the T1DM group but did not reach statistical significance (*p* < 0.05). However, within-group comparisons showed a significant change from supine to upright in SDNN and SD2 for the T1DM group. Overall, these differences may signify a shift towards sympathetic dominance with a concurrent reduction in parasympathetic influence, more prominently observed in the T1DM group compared to the CON group in the upright position. Respiratory rate did not exhibit notable differences within or between the groups.

### Deep breathing and respiratory sinus arrhythmia

Consistent with the findings of Hirsch and Bishop [23] and a review by Shaffer and Ginsberg [11], our study demonstrates that in normally healthy individuals, a slower respiratory rate coupled with a greater tidal volume markedly enhances respiratory sinus arrhythmia, thus increasing HRV compared to normal breathing. This is characterized by an elevation in HR during inspiration, caused by a decrease in parasympathetic activity, and a subsequent decrease of HR during expiration and an increase in parasympathetic activity. Indeed, as has been known for a longer period, deep breathing-induced variations in HR are predominantly influenced by the parasympathetic nervous system [24].

Thus, our results indicate a normal cardiac autonomic response and respiratory sinus arrhythmia to deep breathing in the T1DM group, as evidenced by the HRV indices. Although the groups were matched for V̇O_2max_, age, sex, and BMI, the T1DM group still exhibited a slightly higher resting HR. However, the difference was not statistically significant (median difference 4 bpm, 95% CI − 1.1, 9.2, *p* = 0.082). This observation may align with the research of Wilson et al. [13] and Ewing and Clarke [25], who reported that an elevated HR is connected to early-stage CAN in diabetes. However, HR that is elevated yet responsive to stress by itself is not an indication of clinically significant impairment of the vagal function [2]. Therefore, the deep breathing results suggest that the T1DM group does not exhibit clinically significant changes in cardiac autonomic function.

### Orthostatic test and baroreceptor function

Orthostasis is characterized by a sudden drop in blood pressure upon standing, primarily due to blood pooling in the legs [26]. This physiological change triggers baroreceptors to sense a sudden decrease in blood pressure, consequently initiating a compensatory reflex mechanism. This reflex comprises a rapid decrease in parasympathetic activity and an increase in HR. Concurrently, a more gradual response in sympathetic tone ensues, further elevating HR and enhancing cardiac contractility, vasoconstriction, and activation of the renin–angiotensin system [27]. Therefore, in healthy individuals, a typical physiological response to orthostasis involves a more pronounced decrease in parasympathetic activity and a slight increase in sympathetic activity.

### Supine phase

The consistently higher SBP in the T1DM group might result from sympathetic overactivity, hyperglycaemia-induced vascular maladaptation, and stiffening [28, 29]. Chronic hyperglycaemia, a primary factor in diabetic complications, contributes to hypertension through mechanisms like renin–angiotensin–aldosterone system (RAAS) hyperactivation, sympathetic overactivity, and nitric oxide suppression, leading to myocardial and vascular remodelling [30–32]. These processes also manifest as arterial stiffness, higher stroke volume, and HR [33]. Indeed, the T1DM group showed mean SBP higher than 130 mmHg, which according to European guidelines is considered “high-normal” [34]. However, it is worth noting that our recordings are laboratory measures, as well as blood drawing and anticipation of maximal exercise may elevate sympathetic activity in both our groups. Contrary to prior findings, which observed early CAN detection in T1DM specifically in the supine position using HRV vagal function indices [6], our study found significant HRV differences only in the upright position. However, our T1DM participants had normal cardiorespiratory fitness, no T1DM-related illnesses, and no hypertension; thus, our study populations are not directly comparable.

### Upright phase

A slight decrease in SBP upon standing is a normal response caused by blood pooling in the legs [26]. The consistently higher SBP in the T1DM group may be related to sympathetic overactivity [28]. This is further observed in the HRV indices of SDNN and SD2, which reflect overall autonomic activity and baroreceptor function [11]; these indices were significantly lower from supine to upright positions only in the T1DM group, indicating significant sympathetic activation not observed in the CON group. This activation is also reflected in the significantly increased HR in the upright position in the T1DM group compared to the CON group. In addition to this, the parasympathetic activity in terms of RMSSD and SD1 withdrew significantly more in the T1DM group in the upright position compared to the CON group. Thus, we postulate that the CON group’s SDNN and SD2 results indicate a smaller, more stable sympathetic response to orthostasis compared to the T1DM group.

Parasympathetic activity tends to diminish first in diabetes before further progression of other symptoms related to CAN [35]. However, significantly different parasympathetic modulation between the groups was only observed in orthostasis in our study. This is likely connected to the previously discussed orthostasis-provoked regulation of blood pressure, in which baroreflex-initiated neural excitation causes first a quicker vagal withdrawal and increase in HR before further responses by the sympathetic system such as peripheral vasoconstriction. Significantly higher blood pressure in the T1DM group may require a larger shift from vagal towards sympathetic dominance because of increased cardiovascular tissue stiffness, which is linked to hyperglycaemia in diabetes [28, 30].

On the basis of our findings, we suggest that patients with uncomplicated T1DM and normal cardiorespiratory fitness do not exhibit blunted cardiac autonomic responses to standing, even though blood pressure is significantly higher in the T1DM group compared to the CON group. Chronically elevated blood pressure and altered blood vessel wall mechanics have been associated with baroreceptor resetting, meaning that the threshold for baroreceptor activation in response to elevation in blood pressure increases [36]. This would typically result in a less sensitive HRV response in the T1DM group during both deep breathing and orthostatic testing. However, our deep breathing test results showed no significant differences in respiratory sinus arrhythmia, which is closely associated with baroreceptor function [37], nor in responses to orthostasis, indicating no evidence of baroreceptor sensitivity resetting. Thus, the T1DM group may exhibit a higher level of sympathetic nervous system activity without signs of blunted cardiac autonomic responses, possibly coexisting with effects of increased cardiovascular stiffness and potentially heightened RAAS activity, though cardiovascular stiffness and RAS levels were not measured in this study.

### Limitations

The role of sympathetic overcompensation remains ambiguous, given the complex interplay between sympathetic and vagal function and the indirect measurement methodology of our study. The substantial variation in HRV, combined with our small sample size, has led to wide confidence intervals, potentially affecting the precision of our results. Additionally, short-term HRV is a complex indicator of cardiac autonomic function and is highly sensitive to numerous confounders, necessitating rigorous control. While short-term HRV offers valuable insights into autonomic regulation, it may not fully capture global sympathetic or parasympathetic function. Thus, caution is warranted in interpreting HRV results, as they may not accurately reflect broader autonomic activity across the body.

We acknowledge these limitations and emphasize that HRV should be used thoughtfully within the specific context. We have controlled as many of these factors as feasible, with V̇O_2max_ matching being a novel approach, as lower V̇O_2max_ in T1DM is a common finding. However, we did not control for menstrual cycle phases or inquire about the phase participants were in, which could have influenced HRV results in female participants. Additionally, we did not collect long-term glucose monitoring data, as hypoglycaemic unawareness is more common in patients with long-term diabetes. We must also consider that the supine measures could have been influenced by the blood drawing and the anticipation of maximal exercise testing, as all these procedures were conducted in the same session. Future research should aim for larger sample sizes and better group size balance, given the nature of HRV data. In addition, it would be of interest to investigate the cardiovascular stiffness of the target group with pulse wave velocity, ultrasound, or other imaging techniques, which our study lacked.

## Conclusion

We found distinct, subclinical cardiovascular autonomic nervous system characteristics during orthostatic tests in patients with uncomplicated T1DM. Despite the T1DM group having normal V̇O_2max_, the orthostatic test revealed higher SBP and MAP in both supine and upright positions for T1DM participants, suggesting cardiovascular changes. Additionally, the T1DM group in orthostasis displayed a significant increase in sympathetic activity and a more pronounced withdrawal of parasympathetic activity, as evidenced by the HRV indices. These responses likely serve as compensatory mechanisms against alterations commonly associated with T1DM, such as increased cardiovascular stiffness, elevated blood pressure, and RAAS hyperactivation. However, apart from blood pressure, we did not investigate whether participants had these specific alterations, highlighting the need for further research.

## Supplementary Information

Below is the link to the electronic supplementary material.

Supplementary file1 (DOCX 21 KB)

## Data Availability

The data is not publicly available due to privacy statements. Upon a reasonable request sent to the corresponding author, the data may be partially made available.
