# Supplementary material for: Orthostatic test shows higher systolic blood pressure and sympathetic response in uncomplicated type 1 diabetes patients with normal V̇O2max vs. healthy controls
Source: Clin Auton Res. 2024 Dec 17;35(3):381–91. doi: 10.1007/s10286-024-01094-5 (PMC12137366; doi:10.1007/s10286-024-01094-5)
Supplement: Supplementary file 1 — Supplementary file1 (DOCX 21 KB) [file 10286_2024_1094_MOESM1_ESM.docx]

| **Variable** | **T1DM (n=14)** | **CON (n=31)** | **p-value** |
| --- | --- | --- | --- |
| Total Cholesterol (mmol/L) | 4.29 ± 0.73 | 4.49 ± 0.98 | 0.500 |
| HDL Cholesterol (mmol/L) | 1.90 ± 0.57 | 1.64 ± 0.31 | 0.052 |
| LDL Cholesterol (mmol/L) | 2.16 ± 0.39 | 2.57 ± 0.86 | **0.037** |

Values are presented as mean ± SD

p-values of significant factors (p < 0.05) are presented in bold.
